# Supplementary material for: Cortical feedback loops bind distributed representations of working memory
Source: Nature. 2022 Jul 27;608(7922):381–9. doi: 10.1038/s41586-022-05014-3 (PMC9365695; doi:10.1038/s41586-022-05014-3)
Supplement: Supplementary file 1 — Reporting Summary [file 41586_2022_5014_MOESM1_ESM.pdf]

nature portfolio

Corresponding author(s): Ivan Voitov

Last updated by author(s): May 18, 2022

Reporting Summary

Nature Portfolio wishes to improve the reproducibility of the work that we publish. This form provides structure for consistency and transparency in reporting. For further information on Nature Portfolio policies, see our Editorial Policies and the Editorial Policy Checklist.

Statistics

For all statistical analyses, confirm that the following items are present in the figure legend, table legend, main text, or Methods section.

n/a	Confirmed	
☐	☒	The exact sample size (*n*) for each experimental group/condition, given as a discrete number and unit of measurement
☐	☒	A statement on whether measurements were taken from distinct samples or whether the same sample was measured repeatedly
☐	☒	The statistical test(s) used AND whether they are one- or two-sided<br>*Only common tests should be described solely by name; describe more complex techniques in the Methods section.*
☐	☒	A description of all covariates tested
☐	☒	A description of any assumptions or corrections, such as tests of normality and adjustment for multiple comparisons
☐	☒	A full description of the statistical parameters including central tendency (e.g. means) or other basic estimates (e.g. regression coefficient) AND variation (e.g. standard deviation) or associated estimates of uncertainty (e.g. confidence intervals)
☐	☒	For null hypothesis testing, the test statistic (e.g. *F*, *t*, *r*) with confidence intervals, effect sizes, degrees of freedom and *P* value noted<br>*Give P values as exact values whenever suitable.*
☒	☐	For Bayesian analysis, information on the choice of priors and Markov chain Monte Carlo settings
☒	☐	For hierarchical and complex designs, identification of the appropriate level for tests and full reporting of outcomes
☐	☒	Estimates of effect sizes (e.g. Cohen's *d*, Pearson's *r*), indicating how they were calculated

Our web collection on statistics for biologists contains articles on many of the points above.

Software and code

Policy information about availability of computer code

Data collection	LabVIEW 2013<br>ScanImage 2019
Data analysis	MATLAB 2020a<br>CaImAn (1.8.3)<br>Suite2p (0.7.1)<br><br>The analysis code is publicly available at: www.github.com/ivan-voitov/loops.

For manuscripts utilizing custom algorithms or software that are central to the research but not yet described in published literature, software must be made available to editors and reviewers. We strongly encourage code deposition in a community repository (e.g. GitHub). See the Nature Portfolio guidelines for submitting code & software for further information.

Data

Policy information about availability of data

All manuscripts must include a data availability statement. This statement should provide the following information, where applicable:

- Accession codes, unique identifiers, or web links for publicly available datasets
- A description of any restrictions on data availability
- For clinical datasets or third party data, please ensure that the statement adheres to our policy

The data that support the findings of this study are available from the corresponding author upon reasonable request.

Field-specific reporting

Please select the one below that is the best fit for your research. If you are not sure, read the appropriate sections before making your selection.

☒ Life sciences ☐ Behavioural & social sciences ☐ Ecological, evolutionary & environmental sciences

For a reference copy of the document with all sections, see nature.com/documents/nr-reporting-summary-flat.pdf

Life sciences study design

All studies must disclose on these points even when the disclosure is negative.

Sample size	Data was collected from 23 animals. When possible, given the limitations such as viral vector mediated gene expression, cranial window implant condition, etc., animals were recorded from in multiple sessions. No sample size calculation was performed prior to the start of the experiments. Sample sizes were progressively increased until the onset of the COVID pandemic, when all experiments were halted.
Data exclusions	Animals which entered behavioural training but did not achieve adequate behavioural performance, as measured by trial completion, were excluded from all further study. Animals with cranial window implants which were in poor condition (e.g.exhibited dura regrowth) were excluded from further study.
Replication	Reproducibility was confirmed by having multiple animals in each experimental condition.<br><br>Multi-area optogenetic silencing experiments (Fig. 1) were replicated in 9 mice.<br>Cell-body imaging experiments (Fig. 2 and Fig. 3) were replicated in 7 mice.<br>Axonal imaging and silencing experiments (Fig. 4) were replicated in 7 mice.<br><br>All attempts at replication were successful, given the data exclusion criteria outlined above.<br><br>Preliminary data was collected at another institute in another country (Biozentrum, University of Basel).
Randomization	There were no comparisons across sampled animal populations in this study. All animals used in this study were chosen as the first available from the breeding populations at the local animal facility.
Blinding	There were no explicit control groups in this study (all experimental controls were within-animal).

Reporting for specific materials, systems and methods

We require information from authors about some types of materials, experimental systems and methods used in many studies. Here, indicate whether each material, system or method listed is relevant to your study. If you are not sure if a list item applies to your research, read the appropriate section before selecting a response.

Materials & experimental systems

n/a	Involved in the study	
☒	☐	Antibodies
☒	☐	Eukaryotic cell lines
☒	☐	Palaeontology and archaeology
☐	☒	Animals and other organisms
☒	☐	Human research participants
☒	☐	Clinical data
☒	☐	Dual use research of concern

Methods

n/a	Involved in the study	
☒	☐	ChIP-seq
☒	☐	Flow cytometry
☒	☐	MRI-based neuroimaging

Animals and other organisms

Policy information about studies involving animals; ARRIVE guidelines recommended for reporting animal research

Laboratory animals	All animals were mus musculus, C57BL/6J background. 10 male and 13 female, 8 to 16 weeks in age.<br>9 animals were PV-Cre × Ai32 mice (JAX 017320 and JAX 024109, Jackson Laboratory).<br>1 animal was a wild-type mouse (Charles River).<br>3 animals were Ai-148 × Cux-creER mice (JAX 030328 and JAX 012243, Jackson Laboratory).<br>3 animals were Ai-148 mice (JAX 030328, Jackson Laboratory).<br>7 animals were PV-Cre mice (JAX 017320, Jackson Laboratory).
Wild animals	There were no wild animals used in this study.
Field-collected samples	No field-collected samples were used in this study.

Ethics oversight	All experiments were performed under the UK Animals (Scientific Procedures) Act of 1986, under project license PPL PD867676F, following local ethical approval by the Sainsbury Wellcome Centre Animal Welfare Ethical Review Body.

Note that full information on the approval of the study protocol must also be provided in the manuscript.
